# Mobility impact and methods of diaphragm monitoring in patients with chronic obstructive pulmonary disease: a systematic review

**DOI:** 10.6061/clinics/2020/e1428

**Published:** 2020-01-06

**Authors:** Iramar Baptistella do Nascimento, Raquel Fleig

**Affiliations:** Departamento de Tecnologia Industrial, Universidade do Estado de Santa Catarina, São Bento do Sul, SC, BR

**Keywords:** Chronic Obstructive Pulmonary Disease, Movement, Diaphragm, Reproducibility of Results

## Abstract

The objectives of the study were to identify the factors that limit diaphragmatic mobility and evaluate the therapeutic results of the monitoring methods previously used in patients with chronic obstructive pulmonary disease. The PubMed, Web of Science, Scopus, and LILACS databases were used. A gray literature search was conducted with Google scholar. PRISMA was used, and the bias risk analysis adapted from the Cochrane Handbook for clinical trials and, for other studies, the Downs and Black checklist were used. Twenty-five articles were included in the qualitative synthesis analysis on physiotherapeutic techniques and diaphragmatic mobility. Eight clinical trials indicated satisfactory domains, and on the Downs and Black scale, 17 cohort studies were evaluated to have an acceptable score. Different conditions must be observed; for example, for postoperative assessments the supine position is suggested to be the most appropriate position to verify diaphragm excursion, although it has been shown to be associated with difficulty of restriction and matching in samples. Therefore, we identified the need for contemporary adjustments and strategies that used imaging instruments, preferably in the dorsal position. Therapeutic evidence on the association between the instrumental method and diaphragmatic mobility can be controversial. The ultrasound measurements indicated some relevance for different analyses, for pulmonary hyperinflation as well as diaphragm thickness and mobilization, in COPD patients. In particular, the study suggests that the ultrasound technique with B-mode for analysis and M-mode for diaphragmatic excursion be used with a 2 - 5 MHz with the patient in the supine position. However, the methods used to monitor diaphragm excursion should be adapted to the conditions of the patients, and additional investigations of their characteristics should be performed. More selective inclusion criteria and better matching in the samples are very important. In addition, more narrow age, sex and weight categories are important, especially in patients with chronic obstructive pulmonary disease.

## INTRODUCTION

Pulmonary ventilation is highly dependent on diaphragmatic muscle mobilization, as it is responsible for 70-80% of the movement in the air entering and exiting the airways (1). The diaphragm presents an anatomical composition with a concave face turned toward the abdomen and a convex face in the cephalic direction, providing oscillatory movements that allow variability of the respective diaphragmatic hemidome. During forced expiration, the right dome is in the height of the fourth costal cartilage, while the left dome is at the rib below the cartilage; then, during inspiration, the domes can descend 10 cm (2).

Diaphragm mobility evaluations are used in clinical practice to identify different dysfunctions, so a professional physiotherapist can establish adequate and efficient therapeutic treatment plans in advance (3). Alterations that compromise muscle actions include muscular dystrophies, thoracic and abdominal surgeries, phrenic nerve lesions and chronic obstructive pulmonary diseases (COPD) (4,5). Full diaphragm movements allow effective pulmonary mechanics to function effectively, causing alterations in the anatomical functional structure of the thoracic and abdominal cavity, involving the ideal length-tension relationship and an interactivity with the abdominal musculature.

Patients with COPD present with reduced diaphragmatic muscle mobility and respective dome alterations (6). Such compromises are associated with the obstructive process of the disease. However, although changes in COPD patients are associated with a chest restriction prognosis, pulmonary gas changes, a reduction in inspiratory dynamics or obstructive conditions (6,7), the reduced diaphragm mobility in individuals with COPD seems to present a stronger relationship with air trapping than with hyperinflation or impaired muscle strength (8). This statement is consistent with results in previous studies since researchers have identified the preservation of central neural activity in diaphragmatic dysfunctions of patients diagnosed with the disease (9).

Strategies have been used to visualize and monitor diaphragm dynamics. The main methods are fluoroscopy, which is the gold standard, computed axial tomography, nuclear magnetic resonance, ultrasound and chest X-ray (10-12). Understanding the factors that compromise the diaphragmatic range of action and the methods used with greater reliability on the mobilization of the muscle enables professional physiotherapists to establish a therapeutic strategy that allows both an improvement in muscle action and reliable monitoring. Thus, the present study aimed to identify the factors that limit diaphragmatic mobility and evaluate the results regarding the monitoring methods previously used in patients with COPD.

## METHODS

A systematic review of the literature was conducted. In the course of this research, we used the guidelines in the Preferred Reporting Items for Systematic Reviews and Meta-Analyses (PRISMA) checklist (13). This systematic review was registered in the CRD (Center for Reviews and Dissemination) under the number CRD42019133713.

### Eligibility criteria

According to a pre-established protocol, the methodological characteristics of the studies were collected as follows: a search was conducted for clinical trials, retrospective cohort studies, and prospective, case control and cross-sectional studies with data originating from primary or secondary sources. For this study, we did not consider revision studies, chapters of books, personal articles, editorials, letters, reviews, comments and conference abstracts. For the literary search, the “PICO“ inclusion criteria were (14). The population of interest included COPD patients who underwent diaphragm monitoring and mobilization. The intervention of interest was therapy in studies on the validity and reliability of an imaging instrument and diaphragmatic muscle mobility. Among the studies on instrumental validity, sample data of selected studies and strategies used to compare individuals with COPD and healthy individuals (control) were extracted. The outcomes of interest were those of imaging instruments that were hypothesized to improve the diaphragm mobilization treatment quality in COPD patients and were used according to the conditions, situations and specific characteristics of the patient samples.

### Information sources and collection strategies

The PubMed / MEDLINE, Web of Science, Scopus and LILACS databases were searched. For the search for the first 50 articles in the gray literature, the Google Scholar database was used. The reference lists of the included studies were manually searched to provide a more accurate assessment. Health Science Descriptors (DeCS) of LILACS, a Virtual Health Library, were used to obtain the keywords. The descriptors Pulmonary Disease, Chronic Obstructive; Movement; Diaphragm; and Reproducibility of Results were selected, in association with the Boolean operator “OR&quot;, to obtain articles relevant to the proposed theme.

Consequently, a referential selection process including the following steps was applied for the systematic reviews: the identification of articles from the above-mentioned databases; a screening of the titles and abstracts; an assessment of the population eligibility, methods, and project relevance; an evaluation of the relevance of the data on diaphragmatic and hemidiaphragmatic mobility in COPD patients and the outcomes; an assessment of the studies evaluating factors influencing diaphragmatic dysfunctions in patients with COPD; an assessment of the research on the relationships between diaphragmatic mobility, anthropometric parameters and patient functionality with COPD; and an assessment of reliability and validity the methods used for monitoring diaphragm muscle mobility.

### Studies selection

The two authors extracted the most relevant data from the studies from the databases. Subsequently, the analysis was performed independently, and if there was discord, the two authors reviewed the priorities established in the initial protocol, prioritizing studies with the most recent publication year, the methodology with the broader scope and strong scientific evidence. It is worth noting that when there was any information that was not presented clearly or was missing, the journals authors were contacted for further clarification.

### Data extraction

The authors collected and organized the data and identified the countries in which the qualitative studies that were selected were conducted based on the details in the text. In addition, to facilitate the interpretation of the reader, a table showing the factors that affect diaphragmatic mobility, with the respective strategies and related equipment used in the different studies that were included in the present study, was created, and the details are shown in Table 1.

### Inclusion criteria

Surveys can be administered only to humans. In the analysis of the studies, the techniques and physiotherapeutic strategies used were verified, and high impact research was prioritized. It is noteworthy that both the clinical trial studies and other types of studies assessed the reliability of the instruments and strategies used in patients who were over 18 years old and had COPD. However, studies in individuals with other intercurrences could only be included in the qualitative analysis in the following situations: there was a strong impact on diaphragmatic mobilization, the validity and reliability of methods of diaphragm excursion were analyzed, or relevant factors that could restrict diaphragm excursion were analyzed.

### Exclusion criteria

Nonrelevant scientific articles that addressed conditions that were not focused on diaphragm mobility dysfunctions in patients with COPD or muscle monitoring strategies were excluded. We excluded studies that included other populations with no relation to muscle excursion, had few details in the methods section, had irrelevant study objectives, or had associations in the data that were not elucidated and publications that did not report quantitative results in absolute and relative values. We excluded studies with smokers or individuals with a history of diseases such as cancer or any dysfunctions that could interfere in the different physiotherapeutic tests applied to verify and evaluate diaphragm mobility. Study populations with pregnant women or women with a pregnancy indication were not included.

### Main topics evaluated

- Diaphragm mobility in COPD patients and characteristics that alter diaphragm excursion: interference and factors;
- Diaphragm muscle mobility and methods used: aspects related to the reliability and validity of different strategies and instrumental resources applied.

### Association between measures used

This systematic review considered studies that used proportional analyses with statistical tests to compare results of therapeutics using imaging instruments, the outcomes and the reliability of different strategies. Similarly, regarding the results on diaphragm muscle mobility and/or the intraclass correlation coefficient (ICC) and p value, the significance level differed across the selected studies, as it was either 5% (*p*<0.05) or 1% (*p*<0.01). When the analysis used in the study was not clear, the authors were contacted by e-mail to verify the data were correct. It is worth noting that when a study did not report the *p*-value for the analyses, the confidence intervals were used to determine whether there was statistical significance.

### Assessment of bias risk in the selected studies

The two authors followed the Cochrane Handbook for Systematic Reviews of Interventions (Version 5.1.0) guidelines (15) to assess the clinical trials. They used the adaptation of the tool for bias verification that is shown in table 8.5.d (Cochrane Handbook for Systematic Reviews of Interventions, version 5.1.0, guidelines). The researchers evaluated and considered the results in the following way: a study was considered satisfactory, and of possible allocation, when the study had ≥4 domains shown in the table with a low level of bias. It is worth noting that a study to be selected should present a low risk of bias, particularly in domains 6 and 7; superior studies had a low level of bias in four domains or more, including the sixth and seventh domain. A study was considered unsatisfactory for this study when it had a low risk of bias for only 1, 2 or 3 domains (≤3 domains), as shown in Table 2.

In other types of studies, such as cohort, control and cross-sectional studies, the bias level was assessed by a Downs and Black scale adaptation (16). This scale aims to evaluate studies that are not related to randomized clinical trials. The details regarding the verification methods for diaphragm mobility in different comparisons in patients with COPD were observed. The inclusion criteria regarding the scores were as follows: for a study to be selected, it had to have at least 13 points, regardless of the study type. However, the maximum score was stipulated to be 28 points for the case-control studies according to the scale criteria and 22 points for the cohort and cross-sectional studies. Meta-analyses were not included in this systematic review because of the data heterogeneity between the studies considered, as shown in Table 3.

## RESULTS

### Studies selection

**Figure 1:**
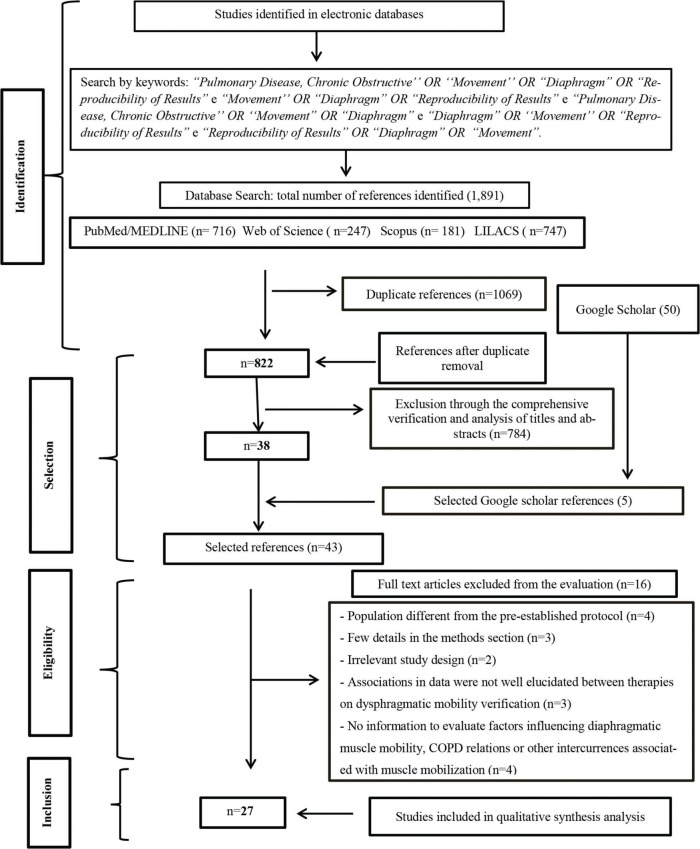
Flowchart of the selection process for the bibliographic search; the diagram is an adapted checklist (PRISMA).

As shown in the flowchart, using the selected databases to search for articles, 1891 studies were identified. After 1069 duplicate articles were removed, 822 articles in English, Portuguese and Spanish were obtained for analysis. A comprehensive title and abstract analysis excluded 784 studies, resulting in 38 articles in the first stage of the study. However, based on the first 50 article analysis referring to the proposed theme, the search with Google scholar allowed the entry of 5 more articles, and a total of 43 studies were eligible for the second stage of the review. In the second stage, the full text of all 43 articles were read, and 16 articles were excluded from the analysis: four (4) were excluded due to the inclusion of a different population from that described in the pre-established protocol, three (3) had few details in the methods section, two (2) had an irrelevant study design, three (3) had poorly elucidated data on the association between therapeutics and dysphragmatic mobility and one (1) had no information of impact to evaluate the factors related to diaphragmatic mobility, COPD or other relevant intercurrences related to the muscle. This study included 27 studies in the qualitative synthesis analysis, as shown in the flowchart in Figure 1.

### General characteristics of the research regarding the countries and study types

The 27 studies selected for the qualitative synthesis were published in the past four decades (1992 to 2019) and were conducted in Brazil - 13 (48.1%), the United States (4) (14.1%) Italy - 4 (14.8%), Turkey - 2 (7.4%), France - 2 (7.4%), Australia - 1 (3.7%) and Canada - 1 (3.7%). Regarding the type of studies selected, there were 10 (37%) clinical trial studies and 17 (63%) cohort studies.

### Absolute data sum regarding the number of searches and punctuation reached

One clinical trial study had low bias risk in six (6) domains, which was the highest proportion of low bias risk, five trials were compatible in five (5) domains and four had four (4) satisfactory domains. Regarding the scores for the adapted Downs and Black scale, seven studies (41.17%) had five (29.4%), three (17.6%) and two (11.7%) points.

### General characteristics about the selected theme outcomes and completed evidence of the present research

The studies reported results on the instruments used and the investigations performed to validate instrumental reliability and the methods used for diaphragmatic mobilization analysis in patients with pulmonary obstructive disease and related intercurrences. In the scientific findings, the need for a wide variety of situations and characteristics that allow the elucidation and conception of therapeutic strategies is realized. Additional adjustments for sample selection appear to be important, and patient baseline characteristics may reduce the potential for confounding effects since the viability of increasing the level of restriction and matching in the samples was observed.

Studies have shown that mobilization performed in the thoracic wall is of fundamental importance for improving diaphragmatic functions. In the scientific findings, the differences between sexes were not statistically significant in right and left hemidiaphragm mobility in COPD patients, and there is a need for further investigations and/or innovative strategies. Surveys were administered to selected study populations of different ages, establishing relevant ranges: between 20 and 30 years old, 60 years old and older, and an average age of 39 years, with a deviation of +/- 13.7 years. Regarding the patient positioning in the different strategies, the supine position was the most commonly used position in the excursion and diaphragm analysis. Regarding the use of equipment, B-mode ultrasound was the most frequently used equipment in the scientific findings. However, M-mode ultrasound indicated higher efficiency, higher reliability, a lower cost and an absence of ionizing radiation. Factors such as the body mass index (BMI) should be further investigated since BMIs corresponding to underweight (<18.5), normal weight (18.5-24.9), overweight (25.0-29.9) and obesity (≥30.0) suggest different complications and restrictions for diaphragm excursion.

## DISCUSSION

### Intercurrences and factors related to diaphragm muscle mobilization in patients with COPD

The alterations that compromise the diaphragmatic muscle action are hypotonic hyperelevation of the muscle, paresis, and lowering and elevation of the hemidome (17-19), which are disorders originating from surgical trauma, infections, tumors and pathologies that affect the nervous and muscular system (20,21).

Air entrapment in the lungs, which leads to hyperinflation and resistance, is one of the factors that characterizes a COPD diagnosis (22). The muscles responsible for inspiration are passively shortened, which generates a condition of mechanical disadvantage, resulting in a compensatory activity of the thoracic wall muscles (8,23,24). Therefore, thoracic wall mobilization allows for diaphragmatic functional improvement and dyspnea relief in individuals diagnosed with COPD (25).

Different physiotherapeutic techniques are used as part of an educational plan, and the aforementioned dysfunctions are associated with dyspnea and exercise intolerance (26,27). Physiotherapeutic treatment involves the professional ability to identify diaphragm mobility reduction regarding the factors that alter the diaphragmatic conditions caused by imprisonment in COPD patients (6,8). The scientific literature presents the performance of physiotherapists evaluating diaphragmatic mobility in individuals with different diseases (12,28).

In a survey of 42 patients without cognitive impairment or neurological disease admitted for cholecystectomy surgery, including 27 (64.3%) females and 15 (35.7%) males, with an age range between 18 and 70 years and an average BMI of 29.4±37.6, the authors identified confidence in the radiological method; however, variability in the diaphragmatic mobility measures was suggested to affect factors related to the age and BMI of patients (12). Another study was conducted with 40 COPD subjects and 40 healthy subjects, whose inclusion criteria for healthy subjects were nonsmokers without a clinical cardiorespiratory diagnosis or neurological disorder, the absence of pregnancy, an FVC and FEV1 of ≥80% of the predicted value and a BMI of <30 kg / m2. The researchers found differences in the mobility of the right hemidiaphragm in relation to the left hemidiaphragm between the men and women but did not find differences between patients with COPD and healthy subjects (*p*>0.05) (29).

In a sample of adults aged 65 years and older who are either patients with combined pulmonary fibrosis (CPFE), idiopathic cystic fibrosis (IPF), and COPD or healthy controls, evaluations of right and left diaphragmatic mobility were performed using fluoroscopy and evaluation instruments with a 0.3 T open MRI system (Hitachi AIRIS I) and ultrasound unit (Mylab30; Esaote; Genoa, Italy) equipped with a 3.2-MHz convex transducer m-mode. All sonographic examinations were performed with subjects in the supine position for the coronal intercostal view and two-dimensional mode for each hemidiaphragm. The results showed a 1% higher percentage of FEV1, FVC%, and RV and a lower TLC in patients with CPFE compared with patients with COPD (30). Another study to evaluate diaphragm movement was conducted by researchers who used magnetic resonance imaging (MRI) fluoroscopy. The objective was to demonstrate a reduction in diaphragm excursion in patients with COPD. The authors selected a sample of individuals of both sexes, who had an average age between the two 56-year-old groups; there were 10 women and 13 men in the COPD group (n=23) with an average diaphragmatic excursion of 20 and 26 mm, respectively, and, in the control group (n=15), there were 6 women and 9 men with an average diaphragmatic excursion of 56 and 69 mm, respectively. All patients were in a supine position and slowly inhaled and exhaled to a maximum capacity for a period of 5 minutes. The patients were analyzed with interrupted respiratory cycle MRI, and differences between the two groups, individuals with COPD and healthy individuals, were noted (*p*<0.05) (31).

Regarding the relationship between an individual’s sex and the diaphragmatic mobility variables, there seems to be no difference in right and left diaphragmatic mobility between healthy men and women. However, among the findings that did not indicate differences between healthy and COPD patients, a disparity was found in the allocation of the men (n=99) and women (n=65) and the absence of spirometric evaluations that confirm the absence of pathologies, which could possibly influence diaphragm mobility (32). Body composition and/or fat and its distribution are factors that present associations, both regarding an increase in the patient's age and the BMI and pulmonary functions that are assessed by spirometry (33).

In a research study published in 2010, a plethysmography exam confirmed movement similarity between individuals of the two sexes in the supine position, even with female inferiority in the respiratory cycle (34). Moreover, spirometric assessments are suggested to be performed before the individuals are included in studies on diaphragmatic mobility, regardless of sex, since there is a strong relationship between muscle mobility and functional aspects related to obstruction and air trapping in the airways (8). Regarding the age of the patient and his or her sex, although no significant statistical impact on the mobility of the right diaphragmatic hemidome was found in comparison with that of the left hemidome, it is important to report, according to the present study findings, that diaphragmatic mobility on both the right and left sides of a man or woman with advanced COPD is lower than that of healthy individuals (8,29). Similarly, in studies by Parreira et al. (34), an influence of age on thoracoabdominal movement and of sex on the respiratory pattern have bene suggested. Other researchers have shown that healthy people with a body mass index (BMI) below the normal standard for younger individuals may show a decrease in muscle movement (32).

### Validity and confidence of the method on diaphragm muscle mobility

Physiotherapists have the ability to interpret and monitor interventions performed in the diaphragm through specific techniques that involve muscle mobilization to improve its fullness of action (3). Among the available resources, the most commonly used resources are instruments that emit electromagnetic radiation (radiography), imaging methods (fluoroscopic) and waves that allow instantaneous captures and imaging (ultrasound).

Fluoroscopy is considered the gold standard and allows the evaluator to interpret diaphragm mobility in real time. However, lateral fluoroscopy is not used if the patient cannot stand or sit, and the quantitative values after evaluation require corrections, which makes this measurement method complex (2,35). With B-mode ultrasound (model Voluson 730 Pro V; GE Healthcare, Amersham, Buckinghamshire, UK), the diaphragmatic muscle can be evaluated directly with the appropriate transducer positioning and indirectly with the probe placed longitudinally or caudally from the skull for right hemidiaphragm analysis only (36); thoracic radiography is easier to perform, has a low cost and seems to be reliable for a direct evaluation of the right and left hemidiaphragmatic mobilization, and intra- and interobserver variability has been observed (12).

Currently, the strategies using the most commonly used radiographs in physiotherapeutic practice to analyze diaphragmatic mobility comprise the distance method (MDdist) and the area method (MDarea). Both consisted of maximal inspiration and maximal expiration at two observation moments in two radiographic images, before and after therapy, to verify the amplitude and function of the diaphragmatic muscular action (36,37).

The distance method (MDdist) allows us to identify the mobility of each hemidiaphragm (HD), which makes it possible to measure the distance between the two highest points between the expiration phases and the maximal inspiration analyzed and evaluated through a longitudinal line that demarcates the hemidome of the same HD. MDdist evaluates muscle mobility in the supine position and anteroposterior incidence, which are more practical positions for patients to assume. On the other hand, the MDarea involves the use of a vegetal paper placed on two overlapping radiographic images on a negatoscope (36).

Patients assume the orthostatic position with posteroanterior incidence, and subsequently, an area design, represented by the diaphragm, is transferred to a software that reports statistical values on diaphragm mobility (37); the statistical values include the two-way ICC as a measure of absolute agreement from a random model and the 95% confidence interval (CI). An ICC higher than 0.70 indicates reliability; values between 0.70 and 0.89 indicate high reliability, and values between 0.90 and 1.00 indicate very high reliability (38,39).

In a study by Saltiel et al. (12), patients with a mean age of 39.7+ / -13.7 years were selected, and those with body mass index (BMI) values above the normal standards (29.4±37.6) were considered overweight. The authors used a radiographic ruler and the MDdist technique to determine the longitudinal thoracic parameter in intra- and interobserver analyses. The endpoint showed a very high ICC for all measurements, i.e., for the radiographic measurements of the right HD (R) and left HD (L) in the intraobserver evaluation (A) (ICC [2.1]=0.99, *p*<0.001 and ICC [2.1]=0.97, respectively *p*<0.001 and for observer B (ICC [2.1]=0.99, *p*<0.001 and ICC [2.1]=0.99 *p*<0.001, respectively). Regarding the analysis of interobserver reproducibility, both the 1^st^ and 2^nd^ evaluation of the right and left hemidiaphragm indicated “equal and very high correlations“ (ICC [2.1]=0.98 and ICC [2.1]=0.99), respectively, *p*<0.001, indicating agreement at the two moments of both cases evaluated with the applied strategies.

In another study (39), the sampling method used was convenience sampling, excluding individuals with a history of cancer, smoking habits, respiratory diseases, or neurological diseases, pregnant women, and noncooperative patients. The patients underwent radiology assessments with a specialized professional. The study selected 43 individuals of both sexes with a mean age of 34±10 years and pulmonary functions within normal limits and used two independent observers (A) and (B) who developed evaluations using the MDdist (36) and MDarea (37) methods at two distinct times within two weeks. Consequently, a third unknown evaluator made the demarcations between the highest and lowest points using a pachymeter according to the moments of maximum inspiration and maximum expiration, respectively, and data were transferred to a software. The study showed “very high“ reliability in both the intraobserver and interobserver analyses, presenting mobility of the HDR and HDL with an ICC>0.90 for both cases (*p*<0.001). The researchers found the MDarea method to be easier to use for assessing diaphragmatic mobility than the MDdist method (39).

Brüggemann et al. (29) compared 40 healthy subjects and 40 people with COPD of both sexes with mean ages of 62.98±7.11 and 66.21±7.8 years, respectively. In the healthy subjects, the inclusion criteria were normal spirometry FVC and FEV1 values ≥80% of the predicted value with a FEV1 / FVC of ≥0.7 and a BMI <30 kg / m2, and they had to be nonsmokers without diagnosis of neurological or cardiothoracic pathologies. On the other hand, individuals with COPD with clinical stability for 30 days before and at the beginning of treatment and no dependence on oxygen were included. The researchers used chest radiography in anteroposterior incidence to assess diaphragmatic mobility. The study did not show a significant difference in the HDD and HDE comparisons between the two groups, and sex seems to be a variable that did not interfere in the position in the dorsal decubitus position.

In contrast, there appears to be a divergence regarding the parameters established in the literature on normal diaphragm mobility (36,40,41), which makes it difficult for statisticians to develop innovative studies and interpret results from contemporary methods. Studies with a strong impact showed divergence in adequate excursion measures of the diaphragm muscle (>20 mm) (40); other studies had values ranging from 23 to 97 mm (40), 25 to 84 mm (32) and even 36 and 92 mm (42). Therefore, it is already well known that both the validity and reliability of the instrument to be applied is essential for clinical practice, and a large error can have consequences that compromise different treatments and professional performance (43).

In the use of ultrasound, regardless of the mode used, success seems to depend on the proper visualization of the diaphragm dome, and certain positions become difficult to determine, such as the position of the ultrasound probe when it is below the costophrenic sinus (40). Therefore, changing positions is critical, and scholars have used B-mode ultrasound (Logic 500, Pro Series^®^; General Electric Medical Systems, Milwaukee, WI, USA) (37) with a 3-convex transducer, 5 MHz, positioned in the right subcostal region in the position of the inferior vena cava, with a perpendicular incidence to the cranial-caudal axis; subsequently, in a field of view to an intraparenchymal portal branch, the position was marked with a cursor during the forced expiration and inspiration. Mobilization of the right diaphragmatic muscle was evaluated with the patient in the right and left lateral position. The authors found that the mobility of the dependent portion of the diaphragm is greater than that of the nondependent portion during spontaneous ventilation, and the change of position was essential to detect muscle mobility variations (44).

Similarly, regarding the use of the equipment and the aspects related to the pathology that overlap diaphragm muscle action impairment, the particularities must be carefully evaluated in individuals such as patients with IPF, for whom mobilization in the diaphragm is lower compared to that in healthy patients. In the study by Boccatonda et al. (45), portable Aloka Prosound ultrasound using 2 - 5 MHz, a convex probe, B-mode (diaphragm examination) and M-mode (diaphragm excursion analysis) was used in subjects who were placed in the supine position. The study indicated differences in muscle excursion between IPF and healthy patients. The statistical results were significant between the two groups (*p*-value <0.001), and the positive correlations between respiratory mobility and normal breathing and the circumference between the two groups were as follows: intervention (*p*=0.032; R=0.37) and control group (*p*=0.034; R=0.614). However, there were no correlations with deep breathing. The accuracy of M-mode ultrasound is noteworthy since it can used in diaphragmatic evaluations with greater precision, greater reliability, a low cost and good reproducibility (42,45). Similar to its discriminative power during deep breathing, the identification of diaphragmatic mobilization among COPD, IPF, CPFE and emphysema patients and normal patients indicates a reduction in diaphragmatic movement in subjects with COPD and IPF (30).

Testa el al. (46) used the A-Hitachi H21 (Tokyo, Japan) USA ultrasound machine with a 210 mm depth, a low scanning speed (10 s per screen) and a 4 MHz convex transducer with patients in the supine position. The same day, a spirometry assessment was performed by an unknown operator using a graphic Cosmed Pony (Rome, Italy). The researchers evaluated the FVC and FEV1. B-mode measurements for the distance from the transducer diaphragm dome across a perpendicular line and M-mode for muscle movement were performed, including as much gain as possible according to the sinusoidal curve (CS) for the measurements. The respiratory rate was measured at the next two peaks of the CS (5 breaths / min), and during resting and forced breathing, the researchers recorded diaphragm muscle excision measurements. Scholars have identified the importance of a safe, noninvasive, low-cost tool for detecting chest diseases, as different methods should be considered to quantify the hemidiaphragmatic position and movement. M-mode scans with the anterior subcostal transverse view are highly viable, fast and reliable solutions.

A survey conducted in 2014 used ultrasound diaphragm thickness measurements to assess associations between ultrasound measurements, respiratory functions, and aspects related to body composition. The researchers used the Mylab 50 Gold Cardiovascular ultrasound system (Esaote Spa, Rome, Italy) and 3.5 to 5 and 7.5 to 12 MHz probes to perform ultrasound measurements. The patients were positioned at a bed inclination of 45° for the procedure. The study showed that diaphragm thickness (TD) is related to lung volume, and both muscle weakness due to loss of fat-free mass (FFM) and body mass index (BMI) were associated with TD. The indexes of pulmonary hyperinflation had relations with DT only in the total pulmonary capacity (TDTLD). Likewise, regarding diaphragm muscle excision, the vital capacity (VC), forced vital capacity (FVC) and exhaled air volume in one second (FEV1) had a strong association with BMI. It is suggested that hyperinflation may be estimated with ultrasound measurements on TDTLC and TLC when adjusted for FFM (47). Loss of fat-free mass (FFM) leads to weakness of skeletal muscles. In patients with COPD, it is one of the main systemic effects of the pathology, and the diaphragm presents a reduced contractile capacity (47). Therefore, physical training becomes indispensable, as it remains the only intervention to reverse some of the underlying skeletal muscle abnormalities, as previously observed in patients with COPD, and a reduction in daily physical activity is the main cause of this development (48). Although the practicality and reliability of the instrument to be applied is preferred, in certain places with greater ease of radiographic device acquisition or when the credibility of the applied analysis method needs to be explored, the current techniques needs to be considered in physiotherapeutic practice since patients differ in their postural abilities; the individual characteristics in the samples should have low heterogeneity and a small possibility of divergence that can interfere in the interpretations of the therapeutic results.

The proper positioning for patients when certain methods are applied can be complex and need to be overcome by professional health care workers who seek the best possible therapeutic monitoring. Thus, the patient's situation may be a limitation for factors, such as postsurgical traumatic strategies and conceptions, diaphragmatic mobilization after cholecystectomy (49), bariatric surgery (50), or even BMI, which has a strong impact on the association between weight and difficulty in respiratory mechanics since it involves large chest compression and suggests differences between the overweight and obesity categories (51). The parameters should be elucidated since a patient’s sex, BMI, waist circumference and age may directly or indirectly affect the strategies to be used (32).

This study has some limitations, such as the small number of studies on the methods used in patients with COPD. Similarly, the demands for sample acquisition, randomization methods, pairings and masking difficulties in the different studies are limitations. The strength of this study was the methods used and associated details, which facilitated the interpretation of the different strategies used by health professionals.

## CONCLUSION

Among other strategies, ultrasound measurements indicated relevance for different analyses, both for pulmonary hyperinflation and for TD and diaphragm mobilization in patients with COPD. In particular, the technique presented by Boccatonda et al. (45) showed relevance; with the patient in supine position, B-mode ultrasound (2 - 5MHz, a convex probe) was used for analysis and M-mode ultrasound was used for the verification of diaphragmatic excursion.

The B-mode method was an efficient and good quality strategy for the analysis of diaphragm excursion, and M-mode method was a reliable and economical method. However, the evidence on therapeutic outcomes regarding the association between the instrumental method and diaphragmatic mobility may be controversial. The methods used to monitor the excursion of the diaphragm must be adapted to the patient's conditions, and additional investigations and predictions about the characteristics and implications in which individuals present themselves should be performed. More selective inclusion criteria are suggested to confirm the instrumental validity and/or predictive values for diagnoses, interpretations and follow-ups. In addition, studies with study populations with more narrow age groups and BMIs specifically categorized as below adequate, normal, overweight or obese for analysis are needed to assess the use of assessment and therapeutic strategies.

## AUTHOR CONTRIBUTIONS

Nascimento IB contributed to the conception of the study, collection and selection of the articles, interpretation and analysis of the results, writing the methods section, data interpretation, design and writing the introduction section. Fleig R contributed to the data collection, study methodology, discussion, and helped writing the results, data collection and study design sections.

## Figures and Tables

**Table 1:** Complications that alter diaphragmatic mobility and the main strategies and instrumental techniques of different scientific research.

Factors affecting diaphragmatic mobility	Strategies and equipment used in different studies to measure diaphragmatic mobility
Dyspnea and exercise intolerance and reduced mobility in patients with COAD	Three studies presented similar techniques for the diaphragm mobilization analysis (3,6,8). Scholars used the ultrasound scanner (Logiq 500, Pro series; GE Medical Systems Milwaukee WI, SA) in B mode and the abdominal motion ratio was employed for the whole body plethysmograph (Collins GS II, Collins, Braintree, MA, USA). A 3,5 MHz convex transducer was positioned on the right subcostal region and on the left portal vein branch. To measure the muscle displacement of the craniocaudal sensor, the scholars placed the patients in dorsal decubitus, and it was measured during expiration and forced inspiration (3). In another study, the method was similar with the patients in dorsal decubitus; diaphragm mobilization was measured according to the displacement of the portal vein intrahepatic branches, using a LOGIQ 500 brand ultrasound system (Pro Series; GE Medical Systems Milwaukee, WI) in B-mode with a 3.5 MHz convex transducer and the field of view of the vein left door demarcated with a cursor at the end of expiration and forced inspiration (6). Another study with the same ultrasound system in B-mode, with a frequency of 3.5 MH and the convex transducer positioned on the right subcostal at an angle of incidence perpendicular to the craniocaudal axis facing the inferior vena cava was conducted. The authors used a whole-body plethysmograph (Collins GS II, Collins, Braintree, MA, USA). The radiologist who identified the left branch of the portal vein from the cursor demarcating movement during expiration and forced inspiration was blinded to patient data (8).
Pulmonary hyperinflation diaphragm COPD Dyspnea	A diaphragmatic mobilization technique yielded the simultaneous observation of the transdiaphragmatic pressure with a difference in pressure between the gastric and esophageal pressures, a change in the diaphragm volume, as measured by lateral fluoroscopy, a duration of inspiration and EMG calculated with 6 pairs of juxtaposed bipolar electrodes, armed in a catheter placed in the lower esophagus. The EMG signals were assessed by the RMS of the signals. The electrically active center of the diaphragm was located in the fourth pair of the electrodes. The subjects were sitting upright, and the measurements were taken at the time of breathing in CRF and during EPAP-induced hyperinflation. The measurements were repeated with an inspiratory threshold (7.5 cm H2O) plus the resistive load (6.5 cm H2O / 1s). The image exposed on the lower chest set a clockwise rotation of 94 °. After the gastric, esophageal and buccal flow pressure were measured, the signals were stored and computerized, and the mouth volume was altered (PowerLab, version 6, ADI Instruments). The authors used lateral fluoroscopic images of the diaphragm and adjacent chest wall, which were stored and analyzed on videotapes (19).
Diaphragmatic plaralysis complication of cardiac surgery	Researchers have verified diaphragmatic mobility through the fluoroscopic &quot;sniff&quot; at the thoracic level with expiratory and inspiratory examinations. For the technique, patients were asked to breathe quickly through the nose for possible detection of both right and left sides of the diaphragm. A radiology expert not involved in the study interpreted the data. Pulmonary functions, as well as its values, were measured by spirometry and plethysmography (SensorMedics 6200, Autobox DL; SensorMedics Corp., Yorba Linda, California) (21).
Hyperinflation and resistance and Dyspnea.	In another study, radiographs were taken of patients in the supine position on a fluoroscopy table. A ruler was used as a parameter for the longitudinal analysis under the trunk region of the subject in the caudal trunk direction for possible subsequent correction of the amplitude provided by the X-ray divergence. The same film was used in all other examinations during an expiratory and inspiratory maneuver, and the method reported by Saltiel et al. (12) was used by the researchers to verify the mobility of the diaphragm in the expiratory and inspiratory phases, i.e., from the highest point of the hemidome, a straight line was drawn, and a pachymer was used (Messen; Sensor Technology Co., Guangdong, China) (22).
Severe airflow obstruction and hyperinflation	Scholars measured the ventilation and muscle activity of the PARA in awake, sitting, and breathing patients (pneumo tachycardus chart and pressure transducer to measure inspiratory airflow). In the expiratory limb, the final CO2 (ETCO2) was continuously verified. CO2-stimulated ventilation was performed by reinhalation of 5.8% CO2 in O2. For the EMG measurement, the electrode pairs of each PARA muscle were preamplified, amplified, rectified, filtered and adjusted with a continuous mean. All signals were monitored in real time on the computer and collected concomitantly for further analysis. The quality and durability of the EMG signal were confirmed by checking the EMG amplitude at the beginning and end of the experiment during maximum inspiration. Pulmonary function tests included spirometry and pulmonary volume and subdivisions by body plethysmography (25).
Dyspnea and air trapping in the lungs that leads to hyperinflation and resistance	A study verified diaphragmatic mobility using the anteroposterior chest radiograph method, using a radiopaque ruler on the longitudinal and craniocaudal throne near the thoracoabdominal transition for possible correction of the rays enlargement and / or divergence. The Wright Respirometer, a British ventilometer, was used as a strategy to ensure maximum volume during diaphragm excursion evaluation. Slow, vital capacity was measured in relation to the near residual volume during expiration, and the inspiratory capacity to total lung capacity was measured. A radiologist performed the exam at both times, for both expiration and inspiration (29).
Idiopathic pulmonary fibrosis	In a survey conducted in 2019, patients underwent clinical examinations, which included measurements of their height, weight, and abdomen and chest circumference. To verify mobilization of the diaphragm, the researchers used a LUS convex ultrasound probe. All patients were evaluated in the supine position with a minimum saturation of S 2> 94%. A subcostal straight ascendant between axillary and middle clavicular area was used. The diaphragm was examined with B-mode ultrasound, and the diaphragm excursions were measured with M-mode ultrasound. The patients underwent 3 measurements in the inspiratory phase, at rest, and deep inspiration (45).
COPD e Body Composition (BMI)	Researchers used a 4 MHz convex probe to measure diaphragm kinetics and thickness. In TD, the measurements were taken from the nearest point where the visibility of the two layers of the diaphragmatic muscle was clearly identified in the right intercostal position. The researchers used the Mylab 50 Gold Cardiovascular ultrasound (Esaote Spa, Rome, Italy) to perform ultrasound measurements using 3.5 to 5 and 7.5 to 12 MHz probes. The position of the patients for the procedure was with the bed inclined 45°. With a 10 MHz probe, the examination was performed with the patient lying in a semirecumbent 45° position. The TD measurements were performed at the end of normal expiration, according to the CRF, after maximal inspiration corresponding to CPT and at the end of maximum expiration according to VR. Thus, the TDFRC, TDTLC and TDRV were evaluated. The operator was the same and proceeded blindly, and the differences between TDTLC and TDFRC were assessed by bioelectrical impedance analysis (47).
Obesity	Diaphragm mobility was performed by chest X-ray in the posteroanterior view of the patient in the standing position. Two radiographic exposures of the same film were taken during complete inspiration and expiration. Scholars used the scanned radiographic image, the area being measured in square centimeters from both the right and left sides, and the axis measured in centimeters. UTHSCSA software, Image Tool for Windows, version 1.28 was used, and the same radiologist performed the analyzes (50).

**Abbreviations:** A.P.N.- Single physiotherapist, BMI- body mass index, COPD - Chronic obstructive pulmonary disease, EMG - Diaphragm electromyogram, EPAP - Positive airway pressure, ETCO 2 - Expiratory limb, end tidal CO2, FEV1- Forced expiratory volume in one second, FVC- Capacidade vital forçada, FRC -Functional residual, MEP - Maximal expiratory pressure, MIP - Maximal inspiratory pressure, capacity, expiratory, IMT- Inspiratory muscle training, PARA - Parasternal intercostal muscle, PEmáx - maximal expiratory pressure, PImáx - maximal inspiratory pressure, RMS - Root mean square, B-mode ultrasound - Emission of high https://drive.google.com/open?id=1WXaSGzCM08k9ehxQECezRQqdhrdSJ8fch frequency so und waves by electrical stimulation of piezoelectric crystals present in the probe or transducer, TD- thickness of the diaphragm, TDFRC- Thickness of the diaphragm at functional residual capacity, TDTLC- thickness of the diaphragm at total lung capacity, TDRV- thickness of the diaphragm at residual volume, TLC- total *lung* capacity, VC- vital capacity.

**Table 2:** Characteristics of the studies included in the qualitative synthesis of the clinical trials and the number of domains met from the adaptation of the Cochrane Handbook bias verification tool.

Author	Year of publication /Sample analyzed	Type of study	Country	Cochrane Handbook DO/ MD	Relative frequency (%)
Yamaguti et al. (3)	2012/n= 30	RCT	Brazil	6/7	85.7
Paulin et al. (6)	2016/ n= 54	CT	Brazil	4/7	57.1
Yamaguti, et al. (8)	2015/n= 74	CT	Brazil	4/7	57.1
Sinderby et al. (9)	2001/n= 10	CT	Canada	5/7	71.4
Kodric et al. (21)	2013/n= 52	RCT	Italy	5/7	71.4
Rocha et al. (22)	2017/n= 50	CT	Brazil	4/7	57.1
Boccadonha et al. (45)	2019/n=24	CT	Itália	5/7	71.4
Smargiassi et al. (47)	2014/n=32	CT	Itália	5/7	71.7
Barbalho-Moulim et al. (50)	2011/n= 32	RCT	Brazil	5/7	71.1
Zerah et al. (51)	1993/n= 46	CT	France	4/7	57.1

Abbreviations: DO - Domain met; MD - Maximum number of domains; CT - Clinical trial; RCT- Randomized clinical trial; n - total number of analyzed samples.

**Table 3:** Characteristics of other studies types included in the qualitative synthesis and scores for the adapted Downs and Black scale.

Author	Year of publication / sample	Type of study	Country	Downs and Black Scale SO / MS	Relative frequency (%)
Ayoub et al. (4)	2001/n=14	Cohort	France	13/22	59.1
Grams et al. (11)	2014/n=41	Cohort	Brazil	16/22	72.7
Saltiel et al. (12)	2013/n=42	Cohort	Brazil	19/22	86.4
Kleinman et al. (17)	2002/n=24	Cohort	EUA	16/22	72.7
Finucane et al. (19)	2005/n=5	Cohort	Australia	17/22	77.3
Martinez et al. (24)	1990/n=45	Cohort	EUA	13/22	59.1
Brüggemann et al. (29)	2018/n=80	Cohort	Brazil	16/22	72.7
Unal et al. (30)	2000/n=38	Cohort	Turkey	16/22	72.7
He et al. (31)	2014/n=124	Cohort	Italy	13/22	59.1
Kantarci et al. (32)	2004/n=164	Cohort	Turkey	13/22	59.1
Parreira et al. (34)	2010/n=104	Cohort	Brazil	16/22	72.7
Leal et al. (35)	2017/n=26	Cohort	Brazil	17/22	77.3
Toledo et at. (36)	2003/n=51	Cohort	Brazil	16/22	72.7
Pedrini et al. (39)	2018/n=43	Cohort	Brazil	17/22	77.3
Gerscovich et al. (41)	2001/n=45	Cohort	EUA	13/22	59.1
Boussuges et al. (42)	2009/n=210	Cohort	EUA	16/22	72.7
Pedrini et al. (49)	2016/n=18	Cohort	Brazil	19/22	86.4

Abbreviations: SO-Score obtained; MS - Maximum score.
